# Hepatoprotective effect of *Herpetospermum caudigerum* Wall. on carbon tetrachloride‐induced hepatic fibrosis in rats

**DOI:** 10.1111/jcmm.13568

**Published:** 2018-04-14

**Authors:** Xin Feng, Ge‐jia Zhong, Da Ji Deng Ba, Bin Yang, Lvyi Chen, Shouying Du

**Affiliations:** ^1^ School of Chinese Materia Medica Beijing University of Chinese Medicine Beijing China; ^2^ Tibetan medicine Institute China Tibetology Research Center Beijing China; ^3^ National Demonstration Center for Experimental Ethnopharmacology Education South‐Central University for Nationalities Wuhan 430074 China; ^4^ School of Pharmaceutical Sciences South‐Central University for Nationalities Wuhan 430074 China

**Keywords:** carbon tetrachloride, hepatic fibrosis, *Herpetospermum caudigerum* Wall., inflammation, Tibetan medicine

## Abstract

Tibetan medicine *Herpetospermum caudigerum* Wall. (HCW) has long been employed to treat hepatitis, inflammatory diseases and jaundice according to the records of “The Four Medical Tantras” in China. This study was investigated to explore the protective effects of HCW on hepatic fibrosis and the possible mechanism in a rat model. Hepatic fibrosis was established by intragastric administration of 3 ml/kg carbon tetrachloride (CCl_4_) twice a week for 6 weeks. CCl_4_‐treated rats were received HCW (1 and 3 g/kg/d) and silymarin (0.1 g/kg/d) from 3 to 6 weeks. The results showed that HCW could significantly decrease the levels of AST, ALT, HA, LN, PCIII, Col IV, TNF‐α, IL‐1β and IL‐6. Moreover, HCW could effectively inhibit collagen deposition and reduce the pathological damage. Analysis experiments finally exhibited that HCW was able to markedly inhibit hepatic fibrosis by modulating the expressions of NF‐κB p65, IκBα, Samd3 and TGF‐β1 proteins. Therefore, our results suggest that HCW has hepatoprotective activity against CCl_4_‐induced hepatic fibrosis in rats by regulating the inflammatory responses.

## 1 INTRODUCTION

Hepatic fibrosis is a serious disease which affects human health around the world.^1^ Evidences suggest that the pathogenesis of hepatic fibrosis is related to the inflammation in activated hepatic stellate cells.^2^, ^3^ During the activation of hepatic stellate cells, plenty of pro‐inflammatory cytokines including interleukin‐6 (IL‐6), IL‐1β and tumour necrosis factor α (TNF‐α) are produced and play important pathogenic roles.^4^ Transforming growth factor‐β1 (TGF‐β1), nuclear factor‐κB (NF‐κB) and multiple growth factors can also promote the activation of hepatic stellate cells and accumulation of extra cellular matrix proteins which contributed to fibrosis.^5^

To control the cirrhosis progression, hepatoprotective drugs are used properly at the stage of hepatic fibrosis. Recently, researchers identify the effective hepatoprotective drugs from herbal medicines due to their low toxicity and free from side effects.

*Herpetospermum caudigerum* Wall. (HCW), a well‐known Tibetan medicine, is used to treat hepatitis, inflammatory diseases and jaundice according to the records of “The Four Medical Tantras” in China. It has the function of clearing heat and regulating “Ci ba dang jiu” in Tibetan medicine theory. Previous studies have showed that HCW is effective on decreasing inflammatory responses and inhibiting lipid peroxidation in CCl_4_‐treated mice.^6^, ^7^, ^8^ On the basis of these researches, our present study is designed to further investigate the hepatoprotective activity of HCW and the effect on modulating NF‐κB and TGF‐β1/Smad signalling pathways in CCl_4_‐induced hepatic fibrosis in rats.

## 2 MATERIALS AND METHODS

### 2.1 Identification of HCW

Herpetospermum caudigerum Wall (HCW) were collected in Tibet of China in October 2016. The materials were identified by Professor Wu Ga, Tibetan academy of Tibetan medicine. A voucher specimen was stored in No. 20161012 at herbarium of Tibetan medicine Institute in China. HCW (5 kg) was extracted by 80% EtOH to obtain HCW extract (501 g). Then, HCW extract was extracted with petroleum ether and ethyl acetate to obtain ethyl acetate fraction (EAF, 100 g).

EAF was identified by HPLC (Welchrom‐Ultimate XB‐C_18_, 4.6 mm × 250 mm, 5 μm) in comparison with reference substances. The detector wavelength was set at 282 nm. The flow rate was 0.8 mL/min, and the injection volume was 2 μL. The mobile phase consists of water containing 50 mmol (w/v) KH_2_PO_4_ (A) and methanol (B) (pH = 3.0). Four reference substances including dehydrodiconiferyl alcohol, herpetrione, herpetin and herpetotriol were used for the qualitative analysis.

### 2.2 Animals and experimental model

Sixty Sprague‐Dawley rats (male, 230‐270 g) were purchased from the Laboratory Animal Center of Huazhong University of Science and Technology (Wuhan, China). All animal experiments were conducted according to the Provision and General Recommendation of Chinese Experimental Animals Administration Legislation. All rats were housed with food and water ad libitum under controlled conditions at 23 ± 2°C with a 12‐h light/dark cycle.

All rats were randomly divided into 5 groups (n = 12 per group): normal control rats and CCl_4_‐treated model rats were orally received 3 ml/kg olive oil and 3 ml/kg CCl_4_ (mixed 1:1 in olive oil; Sigma‐Aldrich, Co., MO, USA) twice a week for 6 weeks and normal saline daily from week 3 to week 6, respectively; rats in treatment groups were orally received 3 ml/kg CCl_4_ twice a week for 6 weeks and silymarin (0.1 g/kg) or HCW (1 and 3 g/kg) daily from week 3 to week 6.

At the end of 6 weeks, rats were killed after overnight fast. Serum samples and liver samples were collected. Some liver tissues immediately were stored at −80°C for analysis, and the others were used for histopathologic examination.

### 2.3 Determination of serum levels of amino transferase (AST), alanine amino transferase (ALT), hyaluronic acid (HA), laminin (LN), type III precollagen (PCIII) and type IV collagen (Col IV)

Serum levels of AST, ALT, HA, LN, PCIII and Col IV were determined according to the manufacturer's instructions of kits (Nanjing Jiancheng Bioengineering Research Institute, Nanjing, China).

### 2.4 Assay of TNF‐α, IL‐1β and IL‐6 in serum and liver tissues

Levels of TNF‐α, IL‐1β and IL‐6 in serum and liver tissues were determined with ELISA kits (Nanjing Key GEN Biotech. Co. Ltd., Nanjing, China) in accordance with the instruction of the manufacturers.

### 2.5 Histopathological examination

Liver tissues were instantly fixed in 10% formalin and processed by routine histopathological procedures. The samples were stained with haematoxylin and eosin (HE) for photomicroscopic assessment.

### 2.6 Immunohistochemical analysis

Liver tissues were fixed in 4% PFA and sectioned in 50 μm/piece. The section was initially permeabilized with 0.4% Triton X‐100 and 1% BSA for 2 hours and then incubated with anti‐TGF‐β1 and anti‐NF‐κBp65 antibodies (Cell Signaling Technology, Beverly, MA, USA) for 72 hours at 4°C. After being washed, sections were incubated with the second antibody for 2 hours at room temperature. Finally, sections were washed, dried and mounted. An arbitrary scope was given to each microscopic field and viewed at a magnification of 200× using a Leica confocal microscope.

### 2.7 Western blot analysis

Liver tissues were homogenized in RIPA buffer with 1% PMSF and phosphatase inhibitor. The equal amount of proteins was separated in 10% SDS‐PAGE gel and then transferred onto PVDF membranes (Millipore, USA). After blocked in 5% BSA, the membranes were incubated with primary antibodies (1:1000; Cell Signaling Technology, Beverly, MA, USA) at 4°C overnight. The membranes were then washed and incubated with the secondary antibodies for 2 hours. Finally, the membranes were exposed to X‐ray film for 1 minute. The relative expression of various proteins was quantified by densitometric scanning using image analysis system.

### 2.8 Statistical analysis

All of the data were presented as mean ± SD. Divergence between groups was compared by one‐way analysis of variance (anova) with Tukey's multiple comparison test. *P* < .05 was considered as significant.

## 3 RESULTS

### 3.1 Identification of HCW

**Figure 1:**
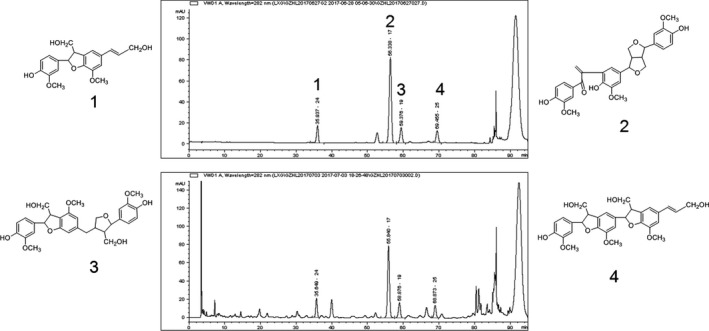
HPLC chromatogram of EAF. (1) Dehydrodiconiferyl alcohol; (2) Herpetrione; (3) Herpetin; (4) Herpetotriol

Four compounds (dehydrodiconiferyl alcohol, herpetrione, herpetin and herpetotriol) were identified by comparing the individual peak retention times with those of the authentic reference standards (Figure 1).

### 3.2 Effect of HCW on biochemical parameters

**Figure 2:**
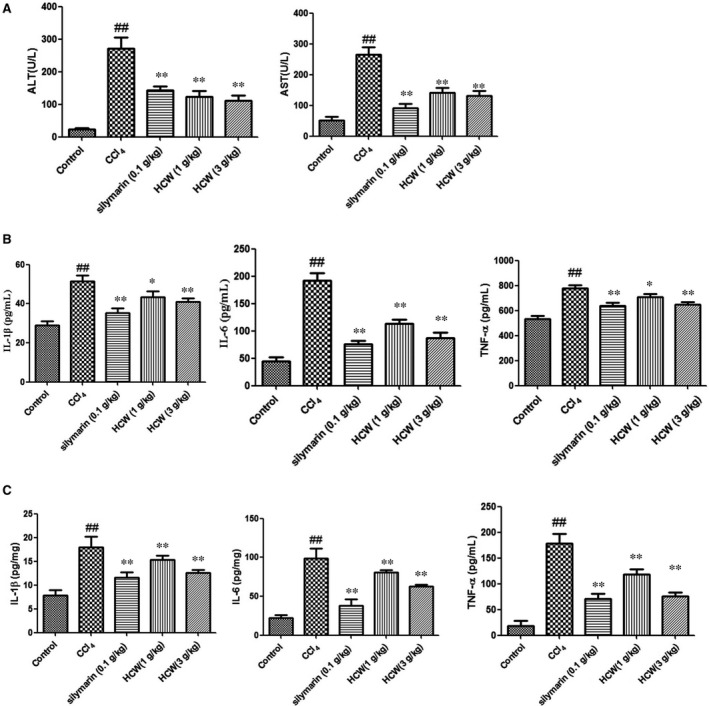
Effect of *Herpetospermum caudigerum* Wall. (HCW) on serum AST and ALT and TNF‐α, IL‐6 and IL‐1β in serum and liver. Values are expressed as mean ± SD. ^#^ *P* < .05, ^##^ *P *< .01 as compared with the normal control group; *P < .05, **P < .01 as compared with the CCl_4_‐treated model group

As illustrated in Figure 2A and Table 1, the serum levels of AST, ALT, HA, LN, PCIII and Col IV in CCl_4_‐treated rats were significantly higher than those of the normal control rats, while treatment with HCW (1 and 3 g/kg) and silymarin (0.1 g/kg) effectively decreased these levels (*P* < .05).

**Table 1:** Effect of *Herpetospermum caudigerum* Wall. (HCW) on serum levels of hyaluronic acid (HA), laminin (LN), PCIII and Col IV

Groups	HA (μg/L)	LN (μg/L)	PCIII (μg/L)	Col IV (μg/L)
Control	49.35 ± 5.26	73.55 ± 13.06	75.57 ± 5.11	58.75 ± 23.14
CCl_4_‐treated model	75.37 ± 22.65###	131.74 ± 20.94###	117.65 ± 29.45###	75.15 ± 14.70#
silymarin (0.1 g/kg)	39.94 ± 12.61***	112.87 ± 16.94*	88.60 ± 6.92**	56.75 ± 15.14**
HCW (1 g/kg)	61.27 ± 8.46*	110.38 ± 27.89*	98.38 ± 10.28*	54.86 ± 16.03*
HCW (3 g/kg)	54.97 ± 8.63**	108.78 ± 6.61**	93.11 ± 10.19**	46.31 ± 10.88***

Values are expressed as mean ± SD. ^#^ *P* < .05, ^##^ *P* < .01, ^###^ *P* < .001 as compared with the normal control group; *P < .05, **P < .01, ***P < .001 as compared with the CCl_4_‐treated model group.

In addition, there were significant increased levels of TNF‐α, IL‐6 and IL‐1β in CCl_4_‐treated rats both in serum and in liver tissues. To the respective, however, the production of TNF‐α, IL‐6 and IL‐1β was effectively inhibited by HCW (1 and 3 g/kg) or silymarin (0.1 g/kg) (Figure 2B and C).

### 3.3 Histopathological findings

**Figure 3:**
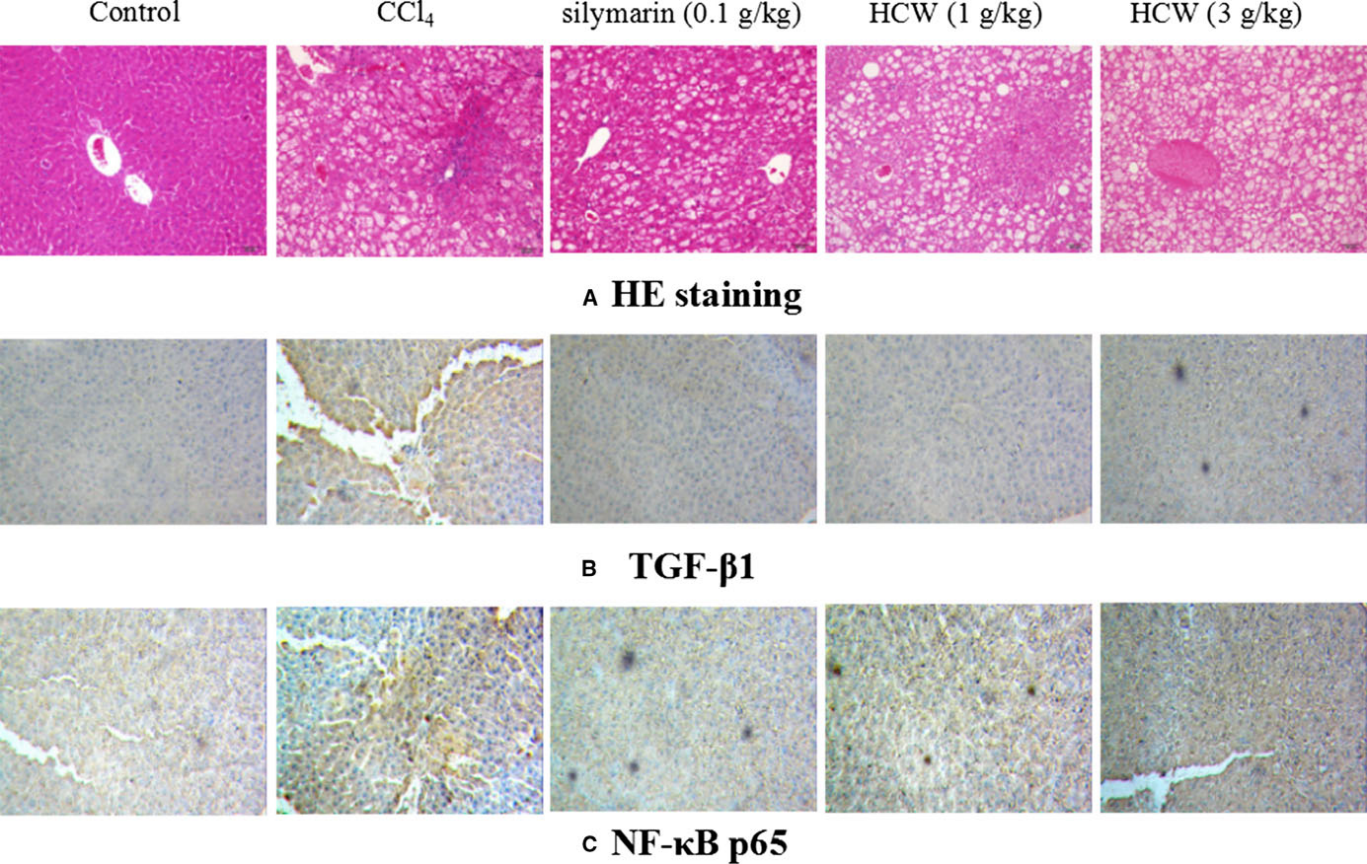
Effect of *Herpetospermum caudigerum* Wall. (HCW) against CCl_4_‐induced hepatic fibrosis in rats. A: HE staining; B and C: Immunohistochemical analysis of TGF‐β1(B) and p‐NF‐κB p65 (C). Magnification: 200 ×

As illustrated in Figure 3A, liver tissue sections in normal rats depicted normal architecture of hepatic central vein and surrounding hepatocytes. In CCl_4_‐treated rats, several changes including ballooning, fat degeneration, necrosis and severe infiltration of inflammatory cells were observed, which demonstrated the successful establishment of hepatic fibrosis. However, HCW or silymarin effectively alleviated the pathological damage with mild ballooning degeneration and few inflammatory cells infiltration.

### 3.4 Immunohistochemical analysis

The expression of TGF‐β1 and NF‐κB p65 proteins in liver tissues was determined by immunohistochemical staining. The expression of TGF‐β1 and NF‐κB p65 proteins was significantly up‐regulated in CCl_4_‐treated rats, while HCW (1 and 3 g/kg) or silymarin (0.1 g/kg) obviously ameliorated these situations (as shown in Figure 3B and C).

### 3.5 Effects of HCW on protein expressions in live tissues of CCl_4_‐treated rats

**Figure 4:**
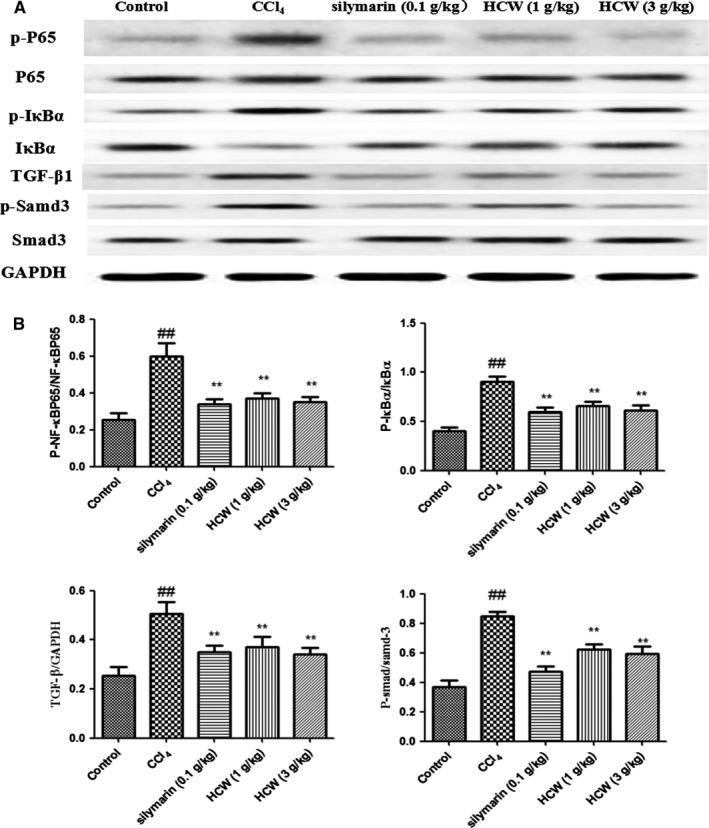
Effects of HCW on inflammation‐related pathways in CCl_4_‐treated rats. Values are expressed as mean ± SD. ^#^ *P* < .05, ^##^ *P* < .01 as compared with the normal control group; *P < .05, **P < .01 as compared with the CCl_4_‐treated model group

The expressions of NF‐κB p65, p‐NF‐κB p65, IκBα, p‐IκBα, TGF‐β1, p‐Samd3 and Samd3 proteins in liver tissues were determined by western blot. The protein expression ratios of p‐IκBα/IκBα and p‐NF‐κB p65/NF‐κB p65 were regulated in liver tissues of CCl4‐treated rats compared with those of normal rats, while HCW (1 and 3 g/kg) or silymarin (0.1 g/kg) exerted suppressive effects on these proteins. Moreover, the up‐regulated expression of TGF‐β1 protein and ratio of p‐Samd3/Samd3 were observed in CCl_4_‐treated rats, while HCW (1 and 3 g/kg) or silymarin (0.1 g/kg) significantly reversed the alterations of TGF‐β1 and p‐Samd3/Samd3 (Figure 4).

## 4 DISCUSSION

Hepatic fibrosis is associated with most chronic liver diseases which results in significant mortality rate around the world. Attentions have been focused on the inflammation‐related pathways as promising therapies for hepatic fibrosis. In the present study, we demonstrated that HCW administration prevented the development of hepatic fibrosis in CCl_4_‐treated rats.

CCl_4_ is a hepatotoxin and has been widely adopted for experimental induction of hepatic fibrosis.^9^ In hepatic fibrosis, the increased levels of AST, ALT, HA, LN, PCIII and Col IV indicate the ability of CCl_4_ to solubilize the lipid components and alter the ion channels of bio‐membranes. In our present study, the hepatic fibrosis was observed with the elevation of serum ALT, AST, HA, LN, PCIII and Col IV in CCl4‐treated rats, while HCW exhibited a significant therapeutic effect on decreasing the levels of those markers. In addition, HCW significantly restored ballooning degeneration and decreased inflammatory cells infiltration in liver tissues. These results indicated the effectiveness of HCW on hepatic regeneration after CCl_4_ damaged.

Inflammatory response is a main event involved in the subsequent fibrogenesis. Upon liver damages, NF‐κB and its downstream signalling are stimulated, which contributes to the activation and survival of hepatic stellate cells.^10^ Evidence indicates that NF‐κB activation is controlled by the degradation of the IκBα. NF‐κB up‐regulates the levels of multiple inflammatory cytokines including TNF‐α, IL‐6 and IL‐1β in the hepatic fibrogenic process. In the present study, HCW obviously decreased the levels of TNF‐α, IL‐6 and IL‐1β and significantly down‐regulated the protein expression ratios of p‐NF‐κB p65/NF‐κB p65 and p‐IκBα/IκBα in CCl_4_‐treated rats. These results suggested that HCW improved the hepatic damage induced by CCl_4_ through regulating NF‐κB signalling to restrict the release of inflammatory mediators.

TGF‐β1 is an important mediator during the activation of hepatic stellate cells. It is ubiquitously distributed and has been recognized as one of most powerful pro‐fibrogenic mediators.^11^ Upon activation, TGF‐β may bind to type I and type II serine/threonine kinase receptors and then activate receptor‐regulated Smads including Smad3.^12^ Previous studies have shown that TGF‐β/Smad signalling pathway inactivation may relieve the hepatic fibrosis.^13^, ^14^ The present study manifested that the higher expression of TGF‐β1 and ratio of p‐Smad3/Samd3 were observed in CCl_4_‐treated rats. HCW remarkably inhibited phosphorylation of Smad3 and activation of TGF‐β1, suggesting that the inhibitory effects of HCW on hepatic fibrosis might be related to its action on hepatic stellate cells deactivation by controlling the TGF‐β1/Smad3 signalling pathway.

However, which is the exert compound that contributes to the effect of HCW on hepatic fibrosis? As reported, dehydrodiconiferyl alcohol inhibits the activation of NF‐jB protein in LPS‐treated macrophage and suggests as an potential agent for the treatment of inflammatory diseases.^15^, ^16^ Herpetrione and herpetin have hepatoprotective effect on CCl4‐treated mice.^17^, ^18^ Pinoresinol downregulated the inflammatory mediators by inhibiting the expression of NF‐κB and AP‐1 proteins in CCl4‐induced liver injury.^19^ On the basis of these above‐mentioned researches, we suggest that the comprehensive effect of these compounds on HCW contributes to the hepatoprotective effect of HCW. Whether other compounds have the hepatoprotective effect or not needs to be addressed in our future research.

In conclusion, the present study demonstrates that HCW has effectively therapeutic activity in the CCl_4_‐induced hepatic fibrosis in rats. The preliminary exploration of the underlying mechanisms exerts the hepatic protection via the modulations of NF‐κB and TGF‐β1/Smad3 signalling pathways. However, further researches are needed for more details in future. Therefore, based on our present study, HCW may be considered as a promising drug for the prevention of hepatic fibrosis.

## CONFLICT OF INTEREST

The authors confirm that there are no conflicts of interest.
